# Impact of a clinical pharmacist on ultrasound-guided venous thromboembolism screening in hospitalized COVID-19 patients: a pilot prospective study

**DOI:** 10.1186/s40780-021-00201-2

**Published:** 2021-04-01

**Authors:** Laura Gillespie, Rashid Z. Khan, John E. Stillson, Connor M. Bunch, Faisal Salim Shariff, Jacob Speybroeck, Anne Grisoli, Meredith Wierman Schmidt, Htay Phyu, Jason Jablonski, Byars Wells, Daniel H. Fulkerson, Lyndsay Oancea, Abraham Leiser, Mark Walsh

**Affiliations:** 1 Department of Quality and Performance Improvement, Saint Joseph Regional Medical Center, Mishawaka, IN USA; 2 Department of Hematology, Michiana Hematology Oncology, Mishawaka, IN USA; 3 South Bend Campus, Indiana University School of Medicine, South Bend, IN USA; 4 Department of Infectious Diseases, St. Joseph Regional Medical Center, Mishawaka, IN USA; 5 Department of Emergency of Medicine, St. Joseph Regional Medical Center, Mishawaka, IN USA; 6 Department of Neurosurgery, St. Joseph Regional Medical Center, Mishawaka, IN USA; 7 Department of Radiology, St. Joseph Regional Medical Center, Mishawaka, IN USA; 8 Department of Internal Medicine, St. Joseph Regional Medical Center, 5215 Holy Cross Pkwy, Mishawaka, IN 46545 USA

**Keywords:** COVID-19, Anticoagulation, Venous thromboembolism, Hypercoagulable, Clinical pharmacist, Ultrasound

## Abstract

**Background:**

The recognition, prevention and treatment of venous thromboembolism (VTE) remains a major challenge in the face of the recent COVID-19 pandemic which has been associated with significant cardiovascular, renal, respiratory and hematologic complications related to hypercoagulability. There has been little literature thus far on the utility of screening ultrasound and the role of the clinical pharmacist in treating these patients.

**Methods:**

We present a prospective pilot program of thirty-one consecutive COVID-19 patients who were provided four extremity screening ultrasounds for VTE on admission. This was coordinated by a clinical pharmacist as part of a multidisciplinary approach. Quantitative and qualitative data were recorded with the goal of describing the utility of the clinical pharmacist in ultrasound screening. Data collected include demographics, information on clinical symptoms or signs at presentation, and laboratory and radiologic results during the hospitalization from each individual electronic medical record.

**Results:**

Nine of the thirty-one patients presented with VTE. Of the nine patients, there were twenty-two total clotted vessels, all of which were asymptomatic. The clinical pharmacist, as the coordinator for a multidisciplinary COVID-19 associated coagulopathy management team, drafted a screening and treatment protocol for anticoagulation prophylaxis and therapy of VTE after ultrasound findings.

**Conclusion:**

VTE screening of hospitalized COVID-19 patients reveals a significant number of asymptomatic VTEs and justifies diagnostic, prophylactic, and treatment measures coordinated by a clinical pharmacist.

## Background

Patients hospitalized due to an acute illness have an elevated risk of venous thromboembolism (VTE) while in-hospital and for up to 45 days after hospitalization [1–9]. Patient survival is reduced significantly among patients with VTE, especially after a pulmonary embolism [10]. The risk of VTE in a patient with an acute infectious disease rises up to 32 times that of the general hospitalized patients in some reports [4, 5, 11]. This risk is elevated in patients hospitalized due to COVID-19, yet the level of risk is not clearly defined. Current laboratory parameters--such as prothrombin time/international normalized ratio, partial thromboplastin time, Clauss Fibrinogen, and D-Dimer--lack sensitivity and specificity for predicting VTE in COVID-19 patients [12–17]. We hypothesize that not only severely ill COVID-19 patients in the intensive care unit (ICU) are at risk of VTE, but also that COVID-19 patients hospitalized to the general floor can develop VTE without clinical signs or symptoms.

To date, there is scant literature regarding the screening of extremities for VTE in COVID-19 patients admitted to an ICU [12–17]. Moreover, there is no current consensus regarding the criteria to predict those patients who require standard, high-dose prophylactic, or full anticoagulation treatment. Therefore, it was decided to embark on a program of four extremity screening ultrasound for all patients admitted to the hospital with documented COVID-19. The coordination, collection, and presentation of data was the responsibility of the clinical pharmacist. The template for this multidisciplinary team in addressing anticoagulation was derived from trauma system models. In such models, the clinical pharmacist’s duties include medication stewardship in addition to the screening, diagnosis, and monitoring of prophylactic and full anticoagulation treatment [18–21]. Coordinating ultrasound screening under the supervision of a clinician gave the clinical pharmacist an additional tool to help guide them in the management of anticoagulation in COVID-19 patients along with daily D-dimer, fibrinogen and other hematologic markers. The clinical pharmacist would be the first to integrate knowledge of a VTE into their care of patients and therefore they readily took on this additional responsibility. Since the start of the COVID-19 pandemic, experience from China, Europe and the United States has revealed that clinical pharmacists are essential for providing event-driven pharmaceutical care which goes beyond routine activities such as medication review, prescription support, and patient counselling [13, 14, 22–25]. Here, the coordination of four-extremity screening ultrasound for all admitted COVID-19 patients was added to the workload of the clinical pharmacist. In addition, the clinical pharmacist was tasked with assisting in the composition, editing and distribution of off-label anticoagulation protocols for prophylaxis and treatment.

Under the supervision of the clinical pharmacist, thirty-one consecutive patients admitted to the general floor and the ICU were screened with four-extremity ultrasound for VTE. Simultaneously the clinical pharmacist, along with a multidisciplinary team, defined the incidence of VTE in our cohort of COVID-19 patients, evaluated clinical and laboratory risk factors, and developed a treatment protocol. A similar screening and treatment protocol development process, headed by their respective clinical pharmacist, may be adopted by all hospitals to provide comprehensive care for the COVID-19 patient.

## Methods

This study included patients with laboratory-confirmed COVID-19 infection who were admitted between 28 April 2020 and 20 May 2020. Clinical specimens were collected from a nasopharyngeal swab, sputum or bronchial aspirate. Diagnosis of COVID-19 infection was made with the reverse-transcriptase–polymerase-chain-reaction assay based on the World Health Organization standard that targets the SARS-CoV-2 E gene and RdRp gene [26]. Thirty-one adults (18 years of age or older) were identified. There were no pregnant women or children admitted to the ICU during the study period. The study protocol was in accordance with the Declaration of Helsinki and approved by the institutional review board of St. Joseph Regional Medical Center Mishawaka. Data collected includes demographics, information on clinical symptoms or signs at presentation, and laboratory and radiologic results during the hospitalization from each individual electronic medical record. All laboratory tests and additional radiologic assessments were performed at the discretion of the attending physician.

A clinical pharmacist was appointed by a multidisciplinary team to coordinate their efforts in identifying and treating COVID-19 patients with VTE. In concert with the infectious disease specialist and hematologist, the clinical pharmacist designed protocols for the ordering of salient laboratory parameters for anticoagulation, as well as criteria for extremity ultrasounds. The clinical pharmacist ordered all ultrasounds of the extremities, initiated and monitored anticoagulation agents, and also assured that all coagulation tests were ordered for each patient according to the defined protocol. Furthermore, the clinical pharmacist, utilizing laboratory and ultrasound findings, made anticoagulant adjustment recommendations.

Upper and lower limb venous compression ultrasound (CUS) was systematically performed in all patients by independent vascular specialists within the first 2 days of admission. The systematic CUS assessed proximal veins. The criterion for establishing the diagnosis of VTE was the lack of compressibility on B-mode ultrasound [27].

## Results

Thirty-one patients were screened during the initial phase from 28 April until 20 May 2020. No patient in whom screening ultrasound detected VTE had overt symptoms defined as edema, erythema, or pain. VTE was identified in nine (29%) patients. All nine (100%) patients with identifiable VTE were asymptomatic. In these nine patients, a total of twenty-two clotted vessels were diagnosed (See Table 1). There were nine VTEs noted in the extremities. None of these patients had a peripherally inserted central catheter (PICC). None of these patients were receiving anticoagulation prior to COVID-19. Of these nine patients with asymptomatic clots, five were admitted to the ICU and all five were intubated. Demographic analysis of the patients is listed in Table 2.

**Table 1:** Localization of VTE on CUS

Anatomic localization of clot	Clots, ***N*** = 22
**Upper extremity**	**15 (68.2%)**
Axillary	2 (9.1%)
Basillic	6 (27.3%)
Brachial	3 (13.6%)
Cephallic	4 (18.2%)
**Lower extremity**	**3 (13.6%)**
Common femoral	1 (4.5%)
Posterior tibial	2 (9.1%)
**Other**	**4 (18.2%)**
Subclavian	1 (4.5%)
Internal jugular	1 (4.5%)
Common iliac	1 (4.5%)
Scrotal	1 (4.5%)

**Table 2:** COVID-19 patient characteristics

	Negative VTE (***n*** = 22)	Positive for VTE (***n*** = 9)	***p***-Value
**Demographics**			
Age (years), mean (SD)	59.0 (17.2)	59.5 (14.5)	–
Female, n (%)	7 (31.8%)	3 (33.3%)	–
BMI (kg/m^2^), mean (SD)	31.3 (9.1)	32.7 (9.1)	–
Prior anticoagulation therapy	0 (0.0%)	0 (0.0%)	–
**Comorbidities, n (%)**			
Hypertension	11 (50.0%)	2 (22.2%)	0.15
Cardiovascular disease	0 (0.00%)	4 (44.4%)	0.0008
COPD	2 (9.09%)	3 (33.3%)	0.10
OSA	1 (4.55%)	1 (11.1%)	0.50
Hepatitis	0 (0.00%)	1 (11.1%)	0.11
Peripheral vascular disease	1 (4.55%)	1 (11.1%)	0.50
Malignancy	1 (4.55%)	1 (11.1%)	0.50
Diabetes	9 (40.9%)	1 (11.1%)	0.10
Renal failure	1 (4.55%)	4 (44.4%)	0.006
Paraplegia	0 (0.00%)	1 (11.1%)	0.11
Singulitis	0 (0.00%)	1 (11.1%)	0.11
**Disposition, n (%)**			
ICU	0 (0.00%)	5 (55.5%)	0.0001
Intubation	0 (0.00%)	5 (55.5%)	0.0001
Death no.	0 (0.00%)	1 (11.1%)	0.11

**Fig. 1:**
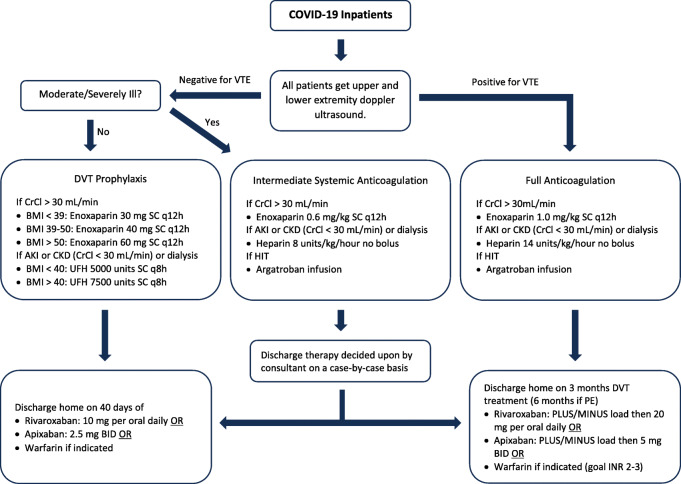
Saint Joseph Regional Medical Center’s Recommended VTE Prophylaxis and Treatment for COVID-19 patients [1, 13, 14]

A protocol for prophylactic and therapeutic anticoagulation was evaluated and revised on a weekly basis by the clinical pharmacist in accordance with guidelines published by the International Society of Hemostasis and Thrombosis. A summary of the current protocol for evaluation and treatment is shown in Fig. 1.

All medication administration was coordinated by the clinical pharmacist. Patients in our study received VTE prophylaxis with daily weight-based subcutaneous enoxaparin or with unfractionated subcutaneous heparin in daily or divided doses. Patients with anatomically proven VTE were treated with weight based subcutaneous enoxaparin, argatroban or with intravenous (IV) unfractionated heparin. Finally, patients admitted to the ICU without anatomic evidence of VTE were treated with either intermediate doses of weight based enoxaparin, argatroban or with therapeutic weight based doses of IV unfractionated heparin.

There were no adverse events that occurred in this group as a result of implementing this program among patients with asymptomatic VTE.

## Discussion

Recent evidence suggests that trauma-induced coagulopathy may be comparable to the hypercoagulable nature of COVID-19 patients [28, 29]. Post-resuscitation trauma patients are at high risk for developing VTE and multiorgan failure due to a shock-induced endotheliopathy and fibrinolytic shutdown. Similar to trauma patients, the mortality and outcome for COVID-19 patients may improve with higher intensity VTE prophylaxis or full anticoagulation to prevent the formation of micro thromboemboli in the pulmonary vasculature [28–42]. COVID-19 coagulopathy, like trauma induced coagulopathy, is characterized by a spectrum of hyper- to hypo-coagulable states in the same patient. Even though our group of patients had no adverse events, hemorrhage as a result of anticoagulation in hospitalized COVID-19 patients was documented. The use of thromboelastography (TEG) for monitoring anticoagulation in hospitalized COVID-19 patients has been shown to be beneficial in guiding changes to anticoagulation for these patients. TEG allows for the clinician to make goal directed anticoagulation decisions on treatment changes which reduces the incidence of hemorrhage [43]. Selection and intensity of anticoagulation in these patients require a dynamic means of evaluation involving screening, prophylaxis, and treatment for VTE [13, 14, 44]. In light of the similar coagulopathies of COVID-19 and trauma patients, it is logical that both patient groups benefit from the expert management of a clinical pharmacist. In our hospital, the clinical pharmacist already coordinated the administration of therapies such as antivirals, convalescent plasma, targeted immunotherapy, and IV antibiotics. The responsibilities of the clinical pharmacist were expanded to include screening and treatment of COVID-19 coagulopathies, including four extremity screening ultrasound for VTE.

Recent data from the NIH ACTIV-4 study demonstrated selective benefit of early anticoagulation for COVID-19 patients. The finding of upper extremity DVTs and/or asymptomatic DVTs would identify higher risk COVID-19 patients in need of anticoagulation early in the admission process. This same trial has demonstrated the potential dangers of delayed anticoagulation in severely ill COVID-19 patients which further increases the importance of early CUS screening and close anticoagulation management of these patients [43, 45]. Nine patients (29%) who received screening ultrasound were found to have asymptomatic VTE despite no PICC line, one of the strongest risk factors for upper extremity clots. Significantly, this supports the hypercoagulopathic state associated with COVID-19 and the need for an evidence-based pharmacologic treatment protocol [8–10, 13, 15, 16, 30, 46]. Recent literature from China, Europe, and the United States has revealed an expanding role for the clinical pharmacist given the evolving nature of guidelines for treating COVID-19 patients [13, 14, 22–25]. Here, the clinical pharmacist drafted guidelines employing ultrasound screening for VTEs to guide anticoagulation therapy; the pharmacist’s role is particularly important with increased off-label use of medications during the COVID-19 pandemic.

Patients who screened positive for VTE were found to have high rates of comorbidities including hypertension, cardiovascular disease, chronic obstructive pulmonary disease and renal failure. This points to a potential adverse additive effect of COVID-19 for those with preexisting medical conditions. Among patients with a negative ultrasound screening for VTE, none were admitted to the ICU or intubated (See Table 2). Five of the nine VTE positive patients diagnosed by ultrasound screening were later admitted to the ICU, and each of those five patients were also intubated. A high incidence of VTE in COVID-19 patients admitted to the ICU has recently been reported [17]. Significantly, this demonstrates the importance of having a clinical pharmacist guide screening and therapy for the complex coagulopathy found in COVID-19 patients. With the findings in this study, the authors hope to prompt similar utilization of pharmacists to prevent clotting or bleeding complications in COVID-19 patients.

## Conclusions

We present a pilot experience of a prospective evaluation protocol for COVID-19 associated VTE coordinated by a clinical pharmacist. This clinical pharmacist facilitated COVID-19 patients’ ultrasound screening, diagnosis, and monitoring of prophylactic and full anticoagulation treatment [18–21]. This multidisciplinary team was adapted from previous programs that utilized a similar dynamic for management of trauma-related anticoagulation prophylaxis and treatment. Nine of thirty-one (29%) COVID-19 patients had radiographic evidence of VTE despite no clinical symptoms on initial presentation. These results confirm the recent emphasis on aggressive VTE prophylaxis and treatment due to the relatively high risk of VTE in COVID-19 [13, 14]. Moreover, these results support the importance of having a clinical pharmacist coordinate screening ultrasound for VTE in COVID-19 patients and management of their anticoagulation. This study demonstrates the dynamic and foundational role the clinical pharmacist can play in the management of coagulopathies in the COVID-19 patient.

## Data Availability

The datasets used and/or analysed during the current study are available from the corresponding author on reasonable request.

## Abbreviations

VTE: Venous thromboembolism
ICU: Intensive care unit
CUS: Compression ultrasound
PICC: Peripherally inserted central catheter
IV: Intravenous
TEG: Thromboelastography
